# Protection by Huang‐Lian‐Jie‐Du decoction and its constituent herbs of lipopolysaccharide‐induced acute kidney injury

**DOI:** 10.1002/2211-5463.12178

**Published:** 2017-01-11

**Authors:** Pei Li, Shan‐Ting Liao, Jun‐Song Wang, Qian Zhang, Ding‐Qiao Xu, Yan Lv, Ming‐Hua Yang, Ling‐Yi Kong

**Affiliations:** ^1^ State Key Laboratory of Natural MedicinesDepartment of Natural Medicinal ChemistryChina Pharmaceutical UniversityNanjingChina; ^2^ Center for Molecular MetabolismNanjing University of Science and TechnologyChina

**Keywords:** Huang‐Lian‐Jie‐Du decoction, metabonomics, qRT‐PCR, septic AKI, western blot

## Abstract

Sepsis, characterized by systemic inflammation, often leads to end‐organ dysfunction, such as acute kidney injury (AKI). Despite of the severity and frequency of septic AKI in clinic, its pathogenesis is still poorly understood. Combined with histopathology evaluations, mortality assessments, biochemical evaluations, reverse transcription (RT) reaction and quantitative real‐time PCR, and western blot, ^1^H NMR‐based metabolomics approach was applied to investigate effects of Huang‐Lian‐Jie‐Du‐Decotion (HLJDD), a traditional Chinese medicine prescription, and its four component herbs on lipopolysaccharide (LPS)‐induced septic AKI and the underlying mechanism. LPS induced kidney dysfunction via activation of NF‐κB and mitogen‐activated protein kinases (MAPKs), by excessive production of IL‐6, tumor necrosis factor‐α, inducible nitric oxide synthase, and COX‐2, producing perturbance in energy metabolism and oxidative stress. HLJDD and its component herbs could effectively inhibit LPS‐induced AKI in mice by inhibiting NF‐κB and MAPK activation and activating the Akt/HO‐1 pathway, and by markedly ameliorating disturbances in oxidative stress and energy metabolism induced by LPS. The four‐component herbs could complement each other.

**Abbreviations**
AKI: acute kidney injury
Akt, HO‐1: hemeoxygenase 1
BUN: blood urea nitrogen
COX‐2: cyclooxygenase 2
Cr: creatinine
CS: citrate synthase
GC‐MS: gas chromatography‐mass spectrometry
GSH: glutathione
GSSG: oxidized glutathione
HLJDD: Huang‐Lian‐Jie‐Du‐Decotion
IL‐6: interleukin‐6
iNOS: inducible nitric oxide synthase
LC‐MS: liquid chromatography‐mass spectrometry
LPS: lipopolysaccharide
MAPKs: mitogen‐activated protein kinases
MDA: malondialdehyde
NF‐κB: nuclear factor‐kappa B
NMR: nuclear magnetic resonance
PK: pyruvate kinase
qRT‐PCR: reverse transcription reaction and quantitative real‐time polymerase chain reaction
SOD: superoxide dismutase
TCA: tricarboxylic acid
TCM: traditional Chinese medicine
TNF‐α: tumor necrosis factor‐α

Sepsis, a clinical syndrome mainly caused by infection, is characterized by systemic inflammation and end‐organ dysfunction. Acute kidney injury (AKI) is common during sepsis development, which has a distinct pathophysiological feature from AKI of nonseptic origin 1. AKI occurs in about half of the patients in septic shock, causing an extremely high mortality 2, 3. Currently, there are no specific effective drugs available to treat human sepsis or septic AKI, due to a vague understanding of the relationships between the inflammatory response and signaling pathways, and end‐organ failure 4. Further investigations on the molecular basis underneath septic AKI should be undertaken to facilitate the development of new therapeutics.

Pathogenesis of sepsis‐induced AKI is due largely to lipopolysaccharides (LPS), the main outer membrane component of Gram‐negative bacteria, which elicited a series of pathological processes. LPS challenge has been one of animal models commonly used to elucidate the mechanisms underlying sepsis‐induced AKI 5. LPS‐induced AKI is associated with severe inflammatory responses, including renal inflammation and renal endothelial dysfunction. Excessive inflammatory responses contribute to the eliciting of acute renal failure. However, the relationship between the inflammatory and metabolic responses was still unknown for sepsis‐induced AKI.

Huang‐Lian‐Jie‐Du‐Decotion is a traditional Chinese medicine (TCM) prescription composed of *Rhizoma coptidis* (RC) (*Coptis chinensis* Franch, Ranunculaceae), *Radix scutellariae* (RS) (*Scutellaria baicalensis* Georgi, Labiatae), *Cortex phellodendri* (CP) (*Phellodendron amurense* Rupr, Rutaceae), and *Fructus gardenia* (*Gardenia jasminoides* Ellis, Rubiaceae) in a weight ratio of 3 : 2 : 2 : 3. As a representative antipyretic and detoxifying TCM formula, HLJDD and its components have been widely acknowledged for their antioxidant, anti‐inflammatory, and antiapoptotic properties 6, 7, 8, 9, 10. Recent studies have indicated the antinephrotoxicity of main components of HLJDD: berberine (main component of RC and CP) exerted protective effects on doxorubicin‐induced nephrotoxicity in mice 11; baicalin (main component of RS) protected mice from kidney injury 12; geniposide (main component of *F. gardenia*) showed its ability of direct binding and neutralization of LPS 13, thus ameliorating LPS‐induced AKI. Although the effects of HLJDD and its individual herb on septic AKI have not been reported to the best of our knowledge.

Metabolomics provides an in‐depth overview of the metabolic status of a complex biosystem at a system level via analytical techniques such as LC‐MS, GC‐MS, and NMR 14, thus simplifying the mechanistic study of complex TCM. With inherent advantages of nonbiased, noninvasive, and easy quantitation, NMR was especially suitable among these techniques for high‐throughput profiling of a complex matrix.

This study used a metabolomic approach, combined with western blot, qRT‐PCR, and chemical test, to profile the metabolic changes at LPS‐induced sepsis in mice and investigated the interventional effects of HLJDD and its herbs. Our results demonstrated that HLJDD and its herbs decrease expression of TNF‐α, COX‐2, HO‐1 and iNOS, GSSG, MDA, BUN and Cr, increase expression of HO‐1 and GSH, and the mechanisms by which these effects occur appear to be through inhibition of the LPS‐stimulated activation of MAPKs and NF‐κB pathways. In addition, HLJDD and its herbs exhibited these efforts by activating Akt/HO‐1 pathway.

## Experimental procedures

### Chemicals and reagents

Lipopolysaccharide (*Escherichia coli*, 055:B5) and deuterium oxide (D_2_O, 99.9%) were bought from Sigma Chemical, Co. (St. Louis, MO, USA). All reagents were of analytical grade.

Huang‐Lian‐Jie‐Du‐Decotion (composed of *R. coptidis*, RS, CP, and *F. gardenia* in a weight ratio of 3 : 2 : 2 : 3) and its constituent herbs [*R. coptidis*, RS, CP, and *Fructus Gardeniae* (GD)] were weighed (each 1 kg) and extracted with 70% ethanol (1 : 10, w/v) under reflux for three times, 1 h each. The extract solutions were combined and lyophilized in vacuum to afford an extract of HLJDD (HD, 256.1 g, yield: 25.61%), RC (256.0 g, yield: 25.60%), RS (488.5 g, yield: 48.85%), CP (200.0 g, yield: 20.00%), and FG (181.7 g, yield: 18.17%), which are dissolved in 0.5% CMC‐Na (carboxymethyl cellulose sodium salt) to the final concentration (according to the ratio in raw medicinal material) of 197 mg·mL^−1^, 46.2 mg·mL^−1^, 69.6 mg·kg^−1^, 10 mg·mL^−1^, and 20 mg·mL^−1^ before intragastrical (i.g.) administration. All herbs were provided by Jiangsu Medicine Company (Nanjing, China, Drug GMP certificate: SUJ0623. Drug Manufacturing Certificate: SUY20110051), and authenticated by Professor Mian Zhang, Department of Medicinal Plants, China Pharmaceutical University, Nanjing, China.

### HPLC‐Q‐TOF‐MS conditions

Chromatographic analysis was performed on an Agilent 1290 series equipped with an Agilent photodiode array detector (Agilent Technologies, Waldbronn, Germany). Mobile phase was composed of two parts: (A) 0.1% formic acid in water; (B) methanol, in a gradient program: 0–4 min, 10% B; 4–15 min, 10–26% B; 15–27 min, 26–28% B; 27–35 min, 28–70% B; 35–55 min, 70–90% B; 55–60 min, 90% B. The flow rate was set at 1 mL·min^−1^ and the injection volume was 8 μL. The HLJDD and its herbs were detected in Fig. S1.

Quadrupole‐Time‐of‐Flight mass spectrometry was performed in the positive and negative mode. The optimal parameters were: gas temperature, 300 °C; drying gas flow rate, 8 L·min^−1^; nebulizer, 35 psig; capillary voltage, 4000 V; capillary current, 6.195 μA; fragmentor, 140 V; skimmer, 65 V; OCT 1 RF Vpp, 750 V. The HLJDD and its herbs were detected in Fig. S2 and compounds are listed in Table S1–S5.

### Ethics statement

All experiments were performed with the approval of the Animal Ethics Committee of the China Pharmaceutical University, and were conducted in accordance with the National Institutes of Health (NIH) guidelines for the Care and Use of Laboratory Animals.

### Animals and treatments

The ICR mice (6–8 weeks; weighing 18–22 g; from the Comparative Medicine Centre of Yangzhou University, Yangzhou, China) were housed in a restricted access room with controlled humidity (50 ± 5%) and temperature (24 ± 2 °C) under alternate cycles of 12 h of light and darkness. Mice were fed with standard mice chow and water *ad libitum* for 1 week to acclimatize with the environment before the start of the study. Mice were then randomly divided into seven groups (each 22): mice in the LPS group (LPS group) received saline solution daily for 7 days before intraperitoneal injection of LPS at 3 mg·kg^−1^; mice in the treatment groups were preadministered with HLJDD, RC, RS, CP, and FG (1 ml per 100 g) once a day for 7 days before intraperitoneal injection of LPS at 3 mg·kg^−1^; mice in the normal control group (NC group) only received the same volume of saline solution daily for 7 days.

Blood was collected from the carotid artery of decapitated mice at 24 h after intraperitoneal injection of LPS, and was then centrifuged at 13 000 ***g*** for 10 min at 4** °**C to obtain serum. Its supernatant was stored at −80** °**C before analysis. Kidney tissues were removed rapidly from the mice after decapitation: the kidney tissues for histological examination were immediately fixed in 10% formalin and embedded in paraffin to be stained with hematoxylin–eosin (HE), and the rest of the tissue samples were immediately stored at −80** °**C.

### Biochemistry

To assess renal function, the concentrations of BUN and CR in serum, and GSH, GSSG, superoxide dismutase (SOD), and MDA in kidney tissues were determined.

### RT‐PCR

The extraction of mRNA in kidney tissues was performed using the RNAiso Plus reagent (TaKaRa Biotechnology Co., Ltd, Dalian, China) according to the manufacturer's protocol. Reverse transcription (RT) reaction and quantitative real‐time PCR were described as previously 15. Quantitation was performed using D cycle threshold method with a LightCycler 480 (Roche Molecular Biochemicals, Mannheim, Germany). Data were normalized to the expression of β‐actin. The values of the target mRNA were normalized according to those of the NC group. The sequences of primers used for quantitative real‐time PCR are listed in Table 1.

**Table 1:** Real‐time PCR primer sequences

Gene	Forward primer sequence	Reverse primer sequence
*β‐Actin*	ACCACACCTTCTACAATGAG	ACGACCAGAGGCATACAG
*TNF‐α*	GACAGTGACCTGGACTGTGG	GAGACAGAGGCAACCTGACC
*IL‐6*	CAGAAGGAGTGGCTAAGGACC	AACGCACTAGGTTTGCCGA
*iNOS*	ATCCATCCCCTGAGCAATGTG	GACCGTCTAATGGGGAGCG
*HO‐1*	AAATGCAATACTGGCCCCCA	GGTGAGGGAACTGTGTCAGG
*COX‐2*	TGAGTGGGGTGATGAGCAAC	TTCAGAGGCAATGCGGTTCT
*PK*	CCGAGATACGCACTGGAGTC	GTGGTAGTCCACCCACACTG
*CS*	TGGTCCCAGGATACGGTCAT	TTGTACAGCTGAGCCACCAG

### Western blot

Protein levels in kidneys were examined by standard western blot. Proteins in kidney tissues were extracted using the Total Protein Extraction Kit (Beyotime, Haimen, Jiangsu, China). The protein concentrations were determined by bicinchoninic acid assay using a Molecular Devices SpectraMax Plus 384 microplate reader (Molecular Devices, Sunnyvale, CA, USA) at 562 nm. Protein samples (50 μg) were separated with 12% or 10% SDS/PAGE and transferred onto poly(vinylidene difluoride) membranes (Bio‐Rad Inc., Hercules, CA, USA). The membranes were blocked with 5% nonfat milk in TBS‐Tween (0.1%) (Junsei Chemical, Japan.) for 2 h and then incubated with monoclonal antibody for β‐actin, Erk1/2 (p44/p42), p‐Erk1/2 (p44/p42) and p38, p‐p38, JNK, p‐JNK, COX‐2, and HO‐1 (1 : 1000 dilution) overnight at 4 °C, followed by secondary antibodies (1 : 10 000 dilution) for 2 h at 25 °C. Immunoreactive protein bands were detected with a ChemiDOC XRS+ (Bio‐Rad, Inc.). Image Lab 4.0 (Bio‐Rad, Inc.) was used to quantitate protein expression based on band intensity.

### Sample preparation for NMR recording

Kidney tissues were weighed (200 mg), homogenized in a mixture of volumetric equivalent acetonitrile and water (2 mL) in an ice/water bath and centrifuged at 13 000 ***g*** for 10 min at 4 °C. The supernatant was collected, lyophilized, and reconstituted in 600 μL of 99.8% D_2_O phosphate (0.2 m Na_2_HPO_4_ and 0.2 m NaH_2_PO_4_, pH 7.0, containing 0.05% sodium 3‐(trimethylsilyl) propionate‐2,2,3,3‐d_4_, TSP). The serum samples were thawed and 300 μL of each was added with 300 μL of additional D_2_O phosphate. After vortexing, tissue and serum samples were centrifuged at 13 000 ***g*** for 10 min to remove any precipitates, the resultant supernatant was then transferred to a 5 mm NMR tube for ^1^H NMR analysis.

### ^1^H NMR spectrometry

The ^1^H NMR spectra of kidney and serum samples were recorded at 25 °C on a Bruker AV 500 MHz spectrometer at 300 K. A 1D NOESYPRESAT pulse sequence for each kidney tissue sample and the transverse relaxation‐edited Carr–Purcell–Meiboom–Gill (CPMG) spin‐echo pulse sequence (RD‐90°‐(τ‐180°‐τ) n‐ACQ) for each serum sample was used to suppress the residual water signal. Prior to Fourier transformation, an exponential window function with a line broadening of 0.5 Hz was used to the free induction decays, which were collected into 32 k data points over a spectral width of 10 000 Hz with an acquisition time of 2.04 s.

### Data processing and analysis

All ^1^H NMR spectra were manually phased, baseline corrected, referenced to TSP (1H, δ 0.00) using Bruker topspin 3.0 software (Bruker GmbH, Karlsruhe, Germany), automatically exported to ASCII files using MestReNova (Version 8.0.1; Mestrelab Research SL, Santiago de Compostela, Spain). ACSII flies were imported into R (http://cran.r-project.org/) and aligned further with an R script developed in‐house. The spectra were adaptively binned between 0.2 and 10 p.p.m. 16. After the removal of resonance due to residual water and its affected regions (4.65–5.25 p.p.m. for kidney extracts) and noisy regions (4.70–9.70 for serum), the integral values of each spectrum were mean‐centered and Pareto‐scaled before multivariate analysis. A supervised orthogonal partial least squares discriminant analysis (OPLS‐DA) was carried out to disclose the metabolic differences between the classes, avoiding being circumvented by an unwanted variation in the data set. A repeated twofold cross‐validation method and permutation test were applied to assess the quality of OPLS‐DA models, whose validity against overfitting was assessed by the parameter *R* ^2^, and predictive ability was described by *Q* ^2^.

Parametric (Student's *t*‐test) or nonparametric Mann–Whitney statistical tests were performed to validate important metabolites that were increased or decreased between groups using R. The threshold for significance was *P* < 0.05 for all tests. Data were expressed as mean ± SD.

## Results

### Mortality

Lipopolysaccharide induced a high mortality (50.0%) of mice in LPS group (11/22), which could be totally avoided by HLJDD treatment (0/22), and decreased by treatments of RC, RS, CP, FG to 9.1% (2/22), 9.1% (2/22), 45.4% (10/22), and 27.3% (6/22).

### Histopathology

**Figure 1:**
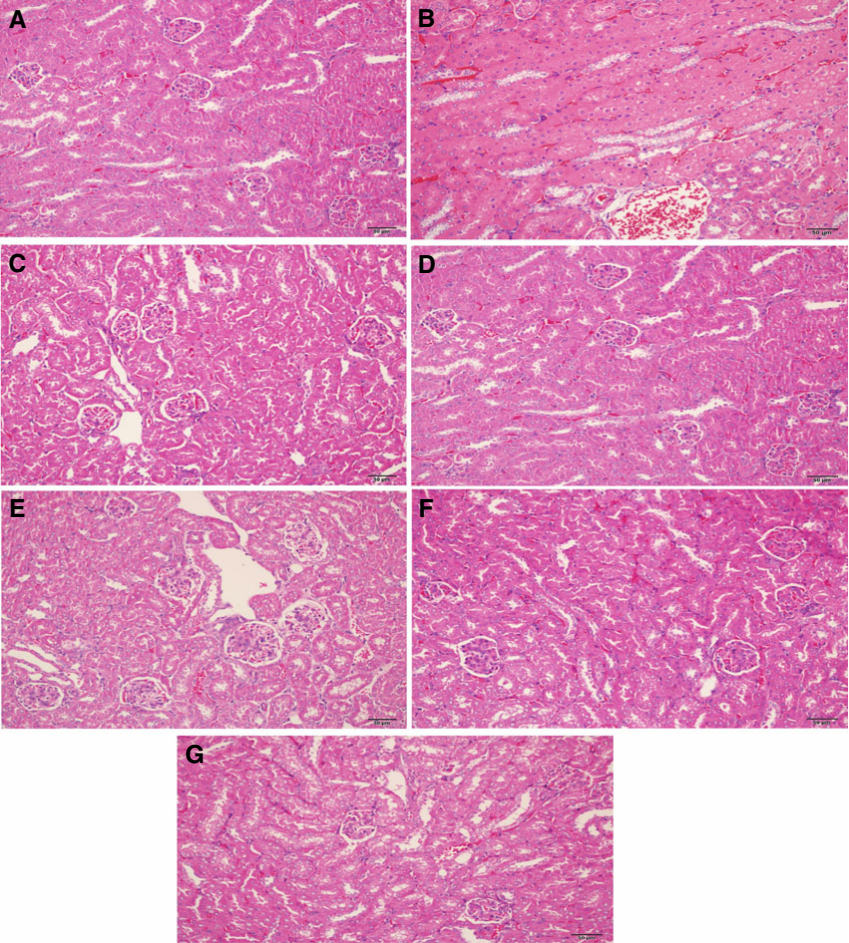
Histopathological photomicrographs of mice kidney sections (A–G) of NC, LPS, HLJDD, RC, RS, CP, and FG groups. The sliced sections were stained with hematoxylin and eosin (H&E), and examined by light microscopy (200 × magnification).

The kidney tissue section of the NC mice showed an apparent normal structure (Fig. 1A) while that of the LPS mice showed significant degeneration and necrosis of tubular epithelial cell and diaphanous tubular cast (Fig. 1B); no significant pathological changes were observed in HLJDD, RC, RS, CP, and FG groups (Fig. 1C–G), which indicated that HLJDD and its component herbs could remarkably alleviate LPS‐induced AKI.

### Biochemistry

**Figure 2:**
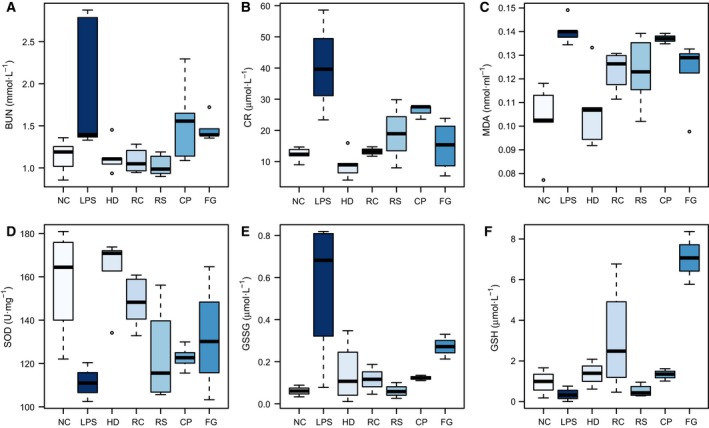
Boxplots for biochemical parameters of BUN (A) and CR (B) in serum; MDA (C), SOD (D),GSSG (E) and GSH (F) in kidney of NC, LPS, HLJDD (HD), RC, RS, CP, and FG groups. The bottom of each box, the line in the box, and the top of the box represent the 1st, 2nd, and 3rd quartiles, respectively. The whiskers extend to 1.5 times the interquartile range (from the 1st to 3rd quartile). All values are mean ± SD (*n* = 5).

Levels of Cr and BUN in serum, GSH, GSSG, SOD, and MDA in kidneys were measured (Fig. 2A–F). The Cr (Fig. 2A) and BUN (Fig. 2B) activities in the LPS group were significantly increased in serum relative to the NC group, suggesting a considerable kidney injury induced by LPS, which could be significantly decreased by HLJDD (HD), RC, RS, CP, and FG treatments. Activities of GSH (Fig. 2F) and SOD (Fig. 2D) in kidneys were obviously decreased as compared with the NC group while levels of MDA (Fig. 2C) and GSSG (Fig. 2E) showed a trend opposite. As again, HLJDD, RC, RS, CP, and FG groups could attenuate these changes in LPS‐induced mice with different emphasis. HLJDD has a much more obvious inhibition on the productions of CR and MDA, and marked augmentation on SOD production than RC, RS, CP, and FG. RC and RS exerted marked inhibitory effects on the levels of BUN and GSSG, comparable to HLJDD. FG has exceptional ability to enhance the GSH level among all groups.

### RT‐PCR

**Figure 3:**
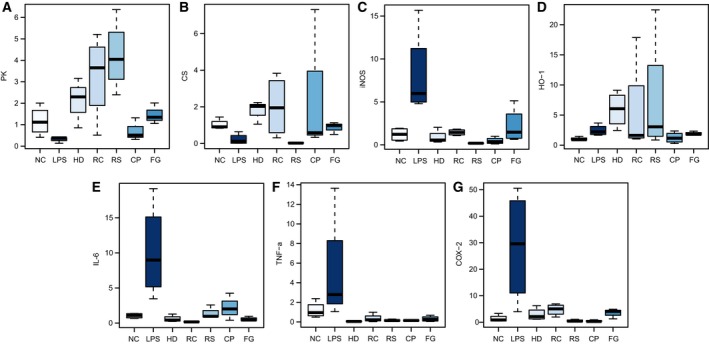
Boxplots for gene expressions of PK (A); CS (B); iNOS (C); HO‐1 (D); IL‐6 (E); TNF‐α (F), and COX‐2 (G) in kidney of NC, LPS, HLJDD (HD), RC, RS, CP, and FG groups. The bottom of each box, the line in the box, and the top of the box represent the 1st, 2nd, and 3rd quartiles, respectively. The whiskers extend to 1.5 times the interquartile range (from the 1st to 3rd quartile). All values are mean ± SD (*n* = 4).

The gene expressions of *pyruvate kinase (PK), citrate synthase (CS), iNOS, HO‐1, IL‐6, TNF‐α, COX‐2* in kidney were determined (Fig. 3A–G). An obvious decrease in *PK* (a regulator of the glucolysis) was observed in the LPS group relative to the NC group (Fig. 3A), suggesting an inhibition of glycolysis after LPS exposure. As a key regulator of the tricarboxylic acid (TCA) cycle, *CS* (Fig. 3B) was markedly decreased after LPS exposure, indicating an inhibited TCA cycle. Both HLJDD (HD) and its component herbs RC, RS, CP, and FG significantly increased the expression of *PK* and *CS*, showcasing their ability to ameliorate LPS‐disturbed energy metabolism. Excessive inflammatory mediators trigger the systemic inflammation and even cause end‐organ damage, sepsis, and death. LPS induced a severe inflammatory response in the body, where *iNOS* and *COX‐2* were potent proinflammatory mediators, and *IL‐6* and *TNF‐α* were key proinflammatory cytokines 17. Significant augmentation in the expressions of *iNOS* (Fig. 3C) and *COX‐2* (Fig. 3G) genes and obvious inhibition of *IL‐6* (Fig. 3E) and *TNF‐α* (Fig. 3F) were observed in mice after LPS exposure as compared with control group, which could be reversed in directions toward the control group, demonstrating marked anti‐inflammatory effects of HLJDD and its component herbs RC, RS, CP, FG, thus alleviating LPS‐induced inflammation damage.

The body also developed self‐protection mechanisms to counteract damage due to excessive inflammatory response, such as HO‐1 18, a cytoprotective enzyme, whose expression was greatly enhanced in mice after LPS exposure. HLJDD and its component herbs further strengthened the increase in the expression of HO‐1 in LPS mice (Fig. 3D), which is favorable for the body to survive the severe crisis induced by LPS. Interestingly, HLJDD group showed no obvious difference in expressions of PK, CS, iNOS, IL‐6, TNF‐α, and COX‐2, but exhibited exceptionally better ability to enhance the expression of HO‐1 than other groups.

### Western blotting

**Figure 4:**
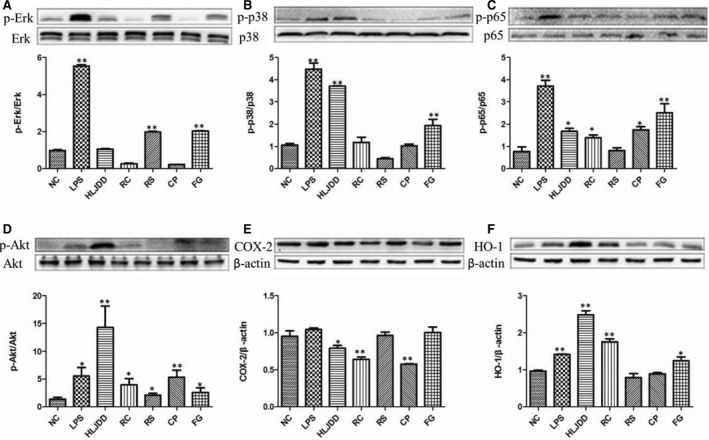
Levels of p‐Erk/Erk (A), p‐p38/p38 (B), p‐p65/p65 (C), p‐Akt/Akt (D), were determined by western blots to investigate effects of HLJDD and its four herbs (RC, RS, CP, FG) on the LPS‐induced AKI. In addition, COX‐2 (E), and HO‐1 (F) protein levels were detected using β‐actin expression as an internal control. *P < 0.05 and ** *P* < 0.01 vs. NC group.

Total kidney lysates were probed with p38, p‐p38, Erk, p‐Erk, JNK, p‐JNK, Akt, p‐Akt, NF‐κB p65, NF‐κB p‐p65, COX‐2, and HO‐1 (Fig. 4). MAPKs (p38 MAPK, JNK, and Erk), NF‐κB, and Akt play important roles in the mediation of inflammatory response 19. Phosphorylation of Erk and p38 was significantly and slightly increased, respectively, in the kidney treated with LPS alone, showing activated MAPK signaling pathway by LPS. Phosphorylation of JNK was not significantly different among all groups (data not shown). As a subunit of the NF‐κB dimer, p65, typically chosen as an index of NF‐κB activation, was obviously activated by LPS. As a result, expressions of COX‐2 were increased in mice administered with LPS, which could be markedly suppressed by treatments of HLJDD and its component herbs by inhibiting LPS‐induced MAPKs and NF‐κB activation. Helpful for the body to counteract LPS‐induced damages 20, phosphorylation of Akt, and the subsequent expression of HO‐1 were significantly increased after exposure to LPS, which were favorably strengthened by the treatments: HLJDD outperformed its component herbs in this regard. Specific effects of individual herbs were found: RC, CP, and RS outperformed other treatments on inhibition of phosphorylation of Erk, p38, and p65, respectively.

### Identification of metabolites in kidney and serum

**Figure 5:**
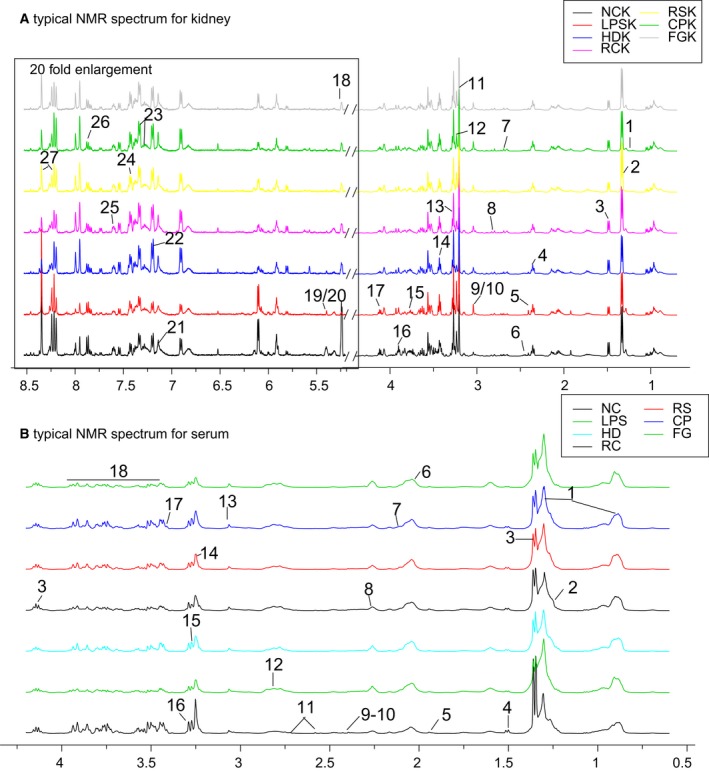
Representative 500 MHz ^1^H NMR spectra of kidney extracts (A) and serum (B) with the metabolites labeled. Because of low signal to noise ratio (SNR), region of (A) in box was enlarged by 20‐fold. Metabolites in kidney extracts: 1. Low‐density lipoprotein or very low density lipoprotein (LDL/VLDL); 2. 3‐hydroxybutyrate (3‐HB); 3. lactate (Lac); 4. alanine (Ala); 5. acetoacetate (Acet); 6. α‐oxoglutarate (2‐OG); 7. sarcosine (Sar); 8. nicotinamide adenine dinucleotide phosphate (NADPH); 9. creatine (Cr); 10. creatinine (Cre); 11. Choline (Cho); 12. phosphocholine (Pco); 13. trimetlylamine oxide (TMAO); 14. taurine (Tau); 15. myo‐inositol (Myo); 16. betaine (Bet); 17. inosine (Ino); 18. lactose (Lact); 19. succinate (Suc); 20. Malate (Mal); 21. (Ans); 22. tyrosine (Tyr); 23. trptophan (Trp); 24. Phenylalanine (Phe); 25. nicotinamide (Nin); 26. uridine (Ude); 27. adenosine (Ade). Metabolites in serum: 1. LDL/VLDL; 2. 3‐HB; 3. Lac; 4. Ala; 5. Ace; 6. *N*‐acetylglucosamine (NAGS); 7. *N*‐acetylglycoprotein (NAGP); 8. *O‐*acetylglycoprotein (OAGP); 9. 2‐OG; 10. pyruvate (Pyr); 11. citrate (Cit); 12. NADPH; 13. Cre; 14; Tau 15. Bet; 16. TMAO; 17. Acet; 18. glucose (Glu).

Representative ^1^H NMR spectra for kidney and serum samples of mice are shown in Fig. 5. Assignments of endogenous metabolites were made by querying publicly accessible metabolomics databases such as Human Metabolome Database (HMDB, http://www.hmdb.ca) and Madison Metabolomics Consortium Database (MMCD, http://mmcd.nmrfam.wisc.edu), and aided by software Chenomx nmr suite 7.5 (Chenomx Inc., Edmonton, AB, Canada) and statistical total correlation spectroscopy (STOSCY) technique. STOCSY technique was adopted to assist metabolite identification and peak integration, which generally encompassed the computation of correlation among the intensities of all peaks in a matrix. STOCSY was calculated and drawn using R language. A total of 27 metabolites in the kidney extracts and a total of 18 metabolites in serum were assigned, consistent with our previous study 21.

### Multivariate analysis of ^1^H NMR spectral data of all groups

**Figure 6:**
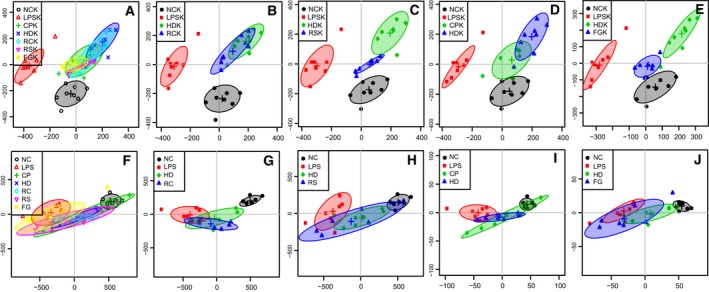
Score plots for OPLS‐DA analysis based on ^1^H NMR spectra of kidney (A–E) and serum (F–J) obtained from the NC, LPS, HLJDD (HD), RC, RS, CP, FG groups.

The kidney and serum 1H NMR data from all groups (Fig. 6A,F) and the NC, LPS, HLJDD (HD), and individual herb group of RC (Fig. 6B,G), RS (Fig. 6C,H), CP (Fig. 6D,I), and FG (Fig. 6E,J) were subjected to OPLS‐DA analysis to compare the treatment effects of HLJDD and its component herbs. Two distinct clusters of groups were observed in the kidney score plots (Fig. 6A–E) where LPS group was located in left regions, far away from NC and treatment groups in the right, demonstrating good performance of HLJDD and its component herbs in rectifying LPS‐induced metabolic disturbance in kidneys. In serum score plots, LPS group was well separated from NC group, with the HD group and other treatment groups in between, overlapped with LPS and NC groups, suggesting that HLJDD and its component herbs could partially ameliorate LPS‐induced metabolic disturbance in serum.

### Metabolic changes in mice treated with LPS and HLJDD

**Figure 7:**
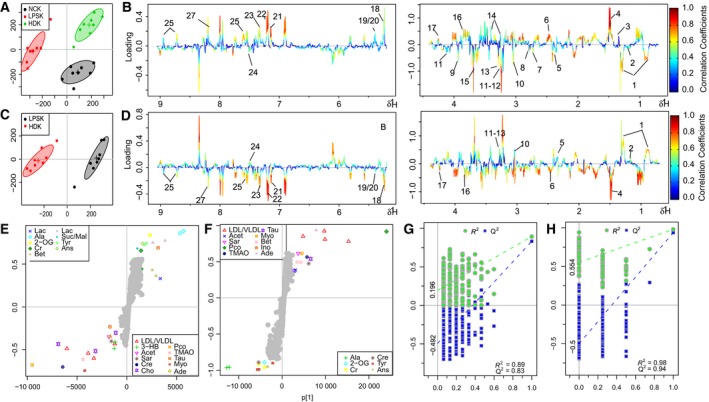
OPLS‐DA analysis of ^1^H NMR data from NC, HLJDD (HD) groups, and LPS group in kidney. (A) Score plot, (B) color‐coded loading plot after removal of water signals and affected regions, (E) S‐plot: OPLS‐DA analysis of ^1^H NMR data from HD groups and LPS group in kidney. (C) Score plot, (D) color‐coded loading plot after removal of water signals and affected regions, (F) S‐plot; OPLS‐DA scatter plot from kidney (G and H) of the statistical validations obtained by 200 times permutation tests.

The OPLS‐DA analysis was performed on the metabolic profiles of NC, LPS, and HLJDD (HD) groups to investigate the therapeutic effects of HLJDD on LPS‐induced AKI. The score plot for kidneys presented a clear clustering of LPS and NC, HLJDD groups (Fig. 7A) with a well goodness of fit (R2Y = 0.89, Q2Y = 0.83) (Fig. 7G) and *P* = 0.001, indicating severe metabolic disturbance in kidney induced by LPS. The S‐plot (Fig. 7E) and loading plots (Fig. 7B) revealed obvious decreases in betaine, taurine, lactate, glucose, and significant increases in 3‐CP, acetoacetate, pyruvate, NADPH, creatine, creatinine, TMAO in LPS mice.

To investigate the direct impact of HLJDD on LPS‐induced AKI, NMR data of LPS and HD groups were subjected to OPLS‐DA analysis. The score plot for kidneys presented a clear clustering of the two groups (Fig. 7C) with a satisfactory goodness of fit (R2Y = 0.98, Q2Y = 0.94) (Fig. 7H) and *P* = 0.016. The S‐plot (Fig. 7F) and loading plots (Fig. 7D) showed amelioration of HLJDD on the disturbed metabolisms in LPS‐induced AKI.

The OPLS‐DA analysis was performed on the metabolic profiles of LPS, NC, and RC groups; LPS, NC and RS groups; LPS, NC, and CP groups; LPS, NC, and FG groups; LPS and RC groups; LPS and RS groups; LPS and CP groups; and LPS and FG groups in kidneys. The score plots, S‐plots, and corresponding loading plots also suggested the amelioration of RC, RS, CP, and FG on the disturbed metabolisms in AKI (data not shown).

The important metabolites differentiating HLJDD vs. LPS, RC vs. LPS, RS vs. LPS, CP vs. LPS, FG vs. LPS in kidneys were further tested for their between‐group difference using univariate analysis, and found to be mostly significant as visualized in the heat map (Fig. 9A) and fold change plots.

**Figure 8:**
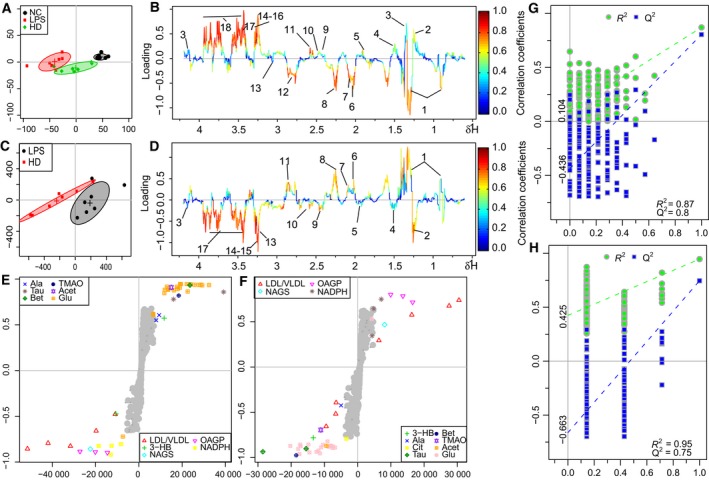
OPLS‐DA analysis of ^1^H NMR data from NC, HLJDD (HD) groups, and LPS group in serum. (A) Score plot, (B) color‐coded loading plot after removal of water signals and affected regions, (E) S‐plot: OPLS‐DA analysis of ^1^H NMR data from HD groups and LPS group in serum. (C) Score plot, (D) color‐coded loading plot after removal of water signals and affected regions, (F) S‐plot; OPLS‐DA scatter plot from kidney (G and H) of the statistical validations obtained by 200 time permutation tests.

The OPLS‐DA analysis was performed on the metabolic profiles of NC, LPS, and HLJDD groups to investigate the therapeutic effects of HLJDD on LPS‐induced AKI. The score plot for serum presented a clear clustering of LPS and NC, HLJDD groups (Fig. 8A) with a well goodness of fit (R2Y = 0.87, Q2Y = 0.8) (Fig. 8G) and *P* = 0.001. The S‐plot (Fig. 8E) and loading plots (Fig. 8B) revealed obvious decreases in 3‐CP, lactate, alanine, acetate, pyruvate, citrate, taurine, betaine, TMAO, acetoacetate, glucose and significant increases in low‐density lipoprotein or very low density lipoprotein, NADPH, creatinine in HLJDD group as compared with LPS group, showing the metabolite turbulence caused by LPS in serum.

To investigate the direct impact of HLJDD on LPS‐induced metabolic disturbance in serum, NMR data of LPS and HD groups were subjected to OPLS‐DA analysis. The score plot for serum presented a clear clustering of these two groups (Fig. 8C) with a well goodness of fit (R2Y = 0.95, Q2Y = 0.75) and *P* < 0.0012. The S‐plot (Fig. 8F) and loading plots (Fig. 8D) revealed amelioration of the metabolic disturbance in serum caused by LPS.

The score plot for serum presented clear clustering of LPS, NC, and RC groups; LPS, NC, and RS groups; LPS, NC, and CP groups; LPS, NC, and FG groups; RC and LPS groups; RS and LPS groups; CP and LPS groups; and FG and LPS groups. The S‐plots and loading plots revealed that RC, RS, CP, and FG could ameliorate LPS‐induced metabolic disturbance in serum (data not shown).

**Figure 9:**
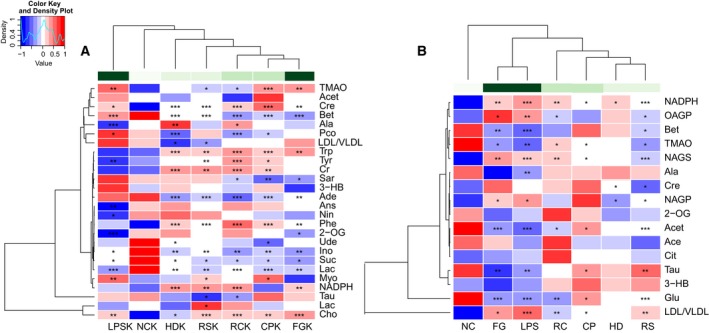
Heatmap visualization of the *z*‐scored levels of metabolites in kidney (A) and serum (B) with stars denoting the differential significance. Row represents metabolites and column represents groups. Color key indicates metabolite quantities value, white: no significant change, deep blue: highest, deep red: lowest, *P* < 0.05 represented statistically significant threshold. *P < 0.05, **P < 0.01 and ***P < 0.001.

The changes of metabolites in serum were visualized by heat map (Fig. 9B) and fold change plots.

## Discussion

**Figure 10:**
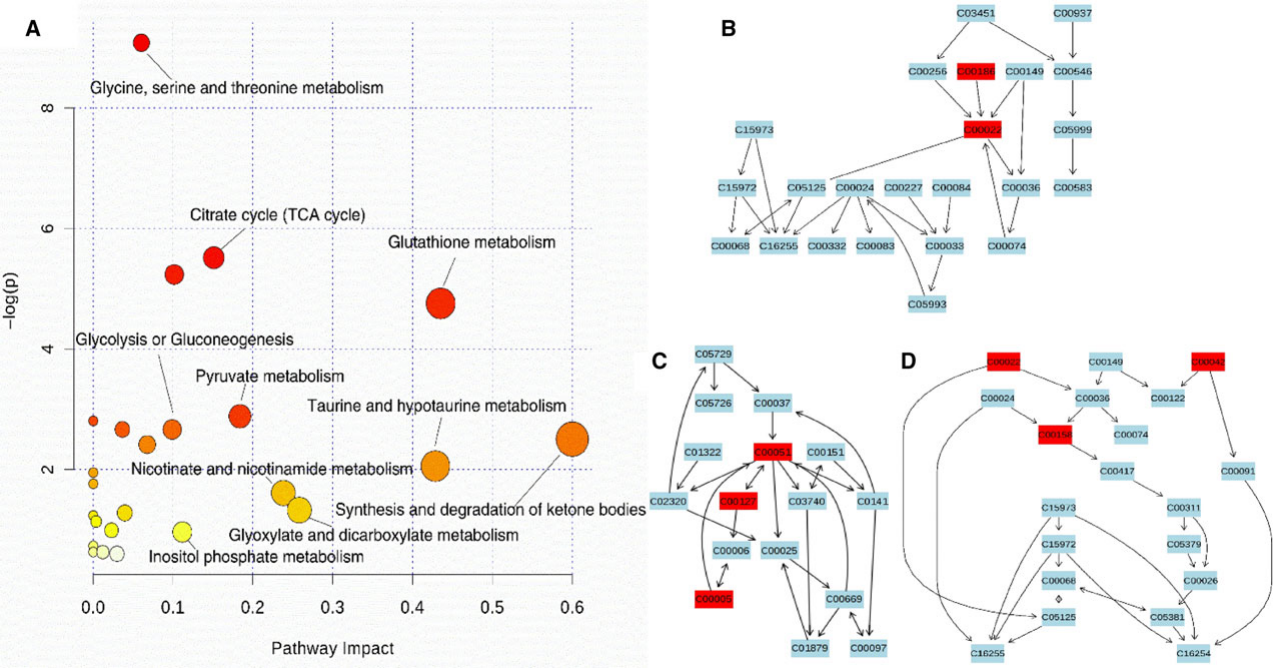
Bubble plots of the altered metabolic pathways of in LPS intoxicated mice compared with the controls and treatment of HLJDD, RC, RS, CP, and FG (A), and the pathway flowchart of the significant affected glutathione metabolism (B), citrate cycle (C), and pyruvate metabolism (D). Bubble area is proportional to the effect on each pathway, with color denoting the significance from highest in red to lowest in white.

**Figure 11:**
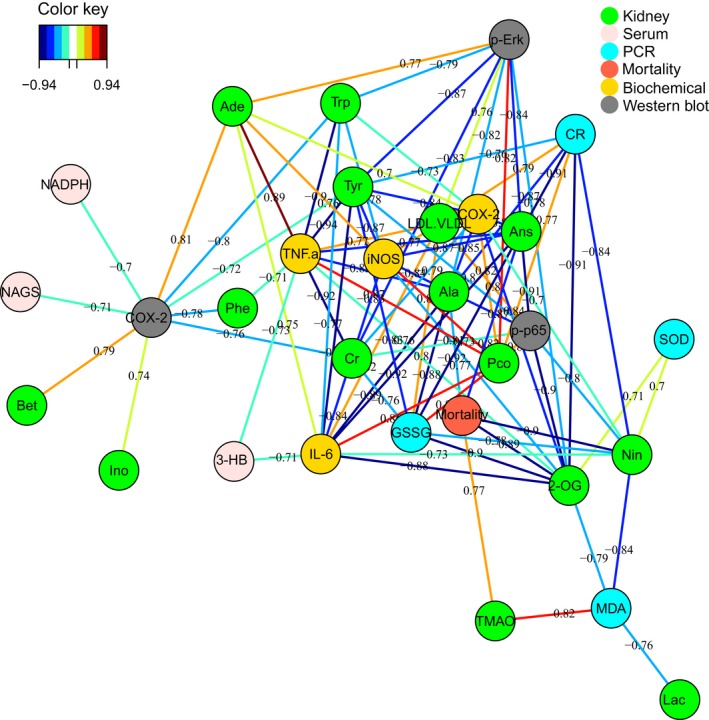
Correlation network determined by canonical (sparse‐partial least‐squares, sPLS) analysis using metabolite concentrations as *x* variables and other parameters as *y* variables. The network is graphically represented with NC, LPS, HLJDD, RC, RS, CP, FG, and biochemical parameters (framed in circles), gene expressions detected by PCR (framed in circles), protein expressions detected by western blot (framed in circles), mortality (framed in circles), and metabolites (framed in rectangles) as nodes, and correlations above a threshold (0.7) as edges (color‐coded according to the correlation coefficients, blue for negative and red for positive correlations).

In our present work, combined with survival rate, histopathological evaluation, biochemical assays, qRT‐PCR, and western blot, 1H NMR‐based metabolomics approach was used to holistically assess therapeutic effect of HLJDD and its component herbs on LPS‐induced AKI in mice. Pathway analysis of the metabolic variations used MetPA on the metabolites that were differentially affected (Fig. 10). The pathways most significantly affected were those for oxidative stress and energy metabolism. Canonical (sparse‐partial least‐squares) analysis of the data 22 was performed and graphical representation of the results (Fig. 11) was generated using a web interface from the University of Queensland (http://mixomics.qfab.org) with metabolite concentrations as *X* variables and the other parameters as *Y* variables to assess the relationships among gene expressions, signal pathways, biochemical parameters, mortality, and metabolic profiles in LPS, HLJDD, RC, RS, CP, and FG mice.

As the most commonly used indicators of for renal function, Cr and BUN were significantly increased in LPS‐dosed mice, suggesting a severe injury of kidneys induced by LPS, which were also supported by where degeneration and necrosis of tubular epithelial cell and diaphanous tubular cast was found in LPS mice. HLJDD and its four component herbs exhibited reversed AKI induced by LPS, which were directly apparent in histopathological inspection, and were also be supported by the significantly decreased nitrogenous waste products (BUN and Cr) detected by serum clinical biochemistry.

Mitogen‐activated protein kinases are crucial in inflammatory responses to LPS exposure by mediating inflammatory signals from the cell surface to the nucleus; that is, Erk, p38, and JNK could activate cytoplasmic enzymes, modulate the activities of other intracellular proteins, and thus phosphorylate and activate various transcription factors, such as NF‐κB‐p65, which induced the transcriptions of proinflammatory mediators, enhancing the expressions of different inflammatory mediators such as TNF‐α, iNOS, and COX‐2 23, 24.

In our study, phosphorylations of Erk and p38, but not that of JNK was found in LPS mice, which together with the obvious increase in NF‐κB‐p65 phosphorylation, suggesting LPS‐activated MAPKs. As a result, marked increases in gene expressions of IL‐6, TNF‐α, iNOS, and COX‐2 were observed in LPS mice, confirming severe inflammatory responses induced by LPS.

Huang‐Lian‐Jie‐Du‐Decotion and its four component herbs inhibited the phosphorylation of p38, Erk, and NF‐kB‐p65, and suppressed the expressions of iNOS, COX‐2, IL‐6, and TNF‐α induced by LPS, which suggested that HLJDD and its component herbs could ameliorate LPS‐induced inflammatory responses by inhibiting MAPK signaling pathway, thus exhibiting anti‐inflammatory protection on LPS‐induced AKI.

The LPS‐induced acute inflammatory responses could be negatively regulated by Akt signaling 25, 26. Akt phosphorylation dampens LPS‐induced NF‐κB activation through direct modification of NF‐κB p65 and then potently suppresses LPS‐induced proinflammatory responses and endotoxic sepsis 27. In addition, Akt phosphorylation could further activate HO‐1 pathway 28, 29, which catalyzes the oxidation of heme to generate potent anti‐inflammatory and antioxidative agents, carbon monoxide, biliverdin, and iron 18. Akt/HO‐1 is highly inducible as an adaptive defense mechanism in response to LPS stimuli. HLJDD and its component herbs significantly strengthened the increasing of Akt phosphorylation and HO‐1 expression, thus alleviating LPS‐induced inflammatory and oxidative injuries in kidney.

Mitogen‐activated protein kinases and NF‐κB could also function as redox‐sensitive transcription factors by activating the overproduction of proinflammatory mediators. Their release increased the generation of reactive oxygen species (ROS), whose accumulation in turn activated MAPKs and NF‐κB, forming a vicious cycle 20, 30, 31.

Several biochemical parameters, including MDA, GSSH, SOD, and GSH, were measured to reflect the status of oxidative stress. Endogenous antioxidant defenses are central to the redox balance in the body. GSH serves as an electron donor to react with ROS, being converted to its oxidized form GSSG. The significant increase in GSSG and obvious decrease of GSH in mice exposed to LPS suggested the overconsumption of GSH to counteract LPS‐induced overgeneration of ROS GSSG could be reduced back to GSH by glutathione reductase in the presence of NADPH 32. NADPH was obviously increased in serum and kidneys of LPS mice, which should be a self‐protection mechanism of the body trying to reduce GSSG to GSH although such an effort was overwhelmed by the overgenerated ROS induced by LPS. The significant decrease in antioxidant enzyme SOD in LPS mice 33, also reflected a severe oxidative status induced by LPS.

During oxidative stress, ROS could cause lipid peroxidation, leading to alterations in membrane permeability and function, and membrane damage 34. Membrane phospholipids, choline, phosphocholine, and ethanolamine, were obviously decreased in LPS mice, suggesting their accelerated utilization to repair damaged cell membrane caused by ROS, which was supported by a marked increase in MDA, a product of lipid peroxidation. The levels of betaine and taurine were significantly decreased in LPS mice. They functioned not only as antioxidant agents but also organic regulatory osmolytes to protect cells from oxidative damage and maintain the structural and functional integrity of membranes 35, 36. In addition, betaine has shown its ability to suppress NF‐κB and inflammatory meditors such as TNF‐α, COX‐2, and iNOS 37. These results demonstrated the overgeneration of ROS induced by LPS, causing severe oxidative stress.

HLJDD and its component herbs significantly lowered the levels of choline, phosphocholine, ethanolamine, MDA, and GSSH in septic mice, and enhanced the levels of GSH, SOD, taurine, and betaine, manifesting their ability to counteract ROS and ameliorate the status of oxidative stress, which could be ascribed to their inhibition on MAPKs and NF‐κB, and activation of Akt.

The ROS and proinflammatory mediators could both damage mitochondria, the major source of ROS, releasing more ROS, thus producing a vicious cycle 30. As the major site of energy production, mitochondrial damage would lead to insufficient energy supply 38. PK, glucose, lactate, alanine, citrate, and OG were significantly decreased, ketone bodies [3‐hydroxybutyrate (3‐HB), acetoacetate], creatine and creatinine, and breakdown products of ATP (adenosine and inosine) were obviously increased in LPS mice, demonstrating a status of energy deficiency induced by LPS.

Significant increases of lactate and alanine (anaerobic products of pyruvate) in serum and kidneys were observed in LPS‐dosed mice after treatments with HLJDD and its component herbs, suggesting an enhanced glycolysis, which was also verified by the obviously increased expressions of PK, a regulator of glycolysis. The treatments also markedly increased the serum levels of glucose, thus increasing energy availability. As the major means of energy production, the TCA cycle in LPS mice was enhanced by HLJDD and its four component herbs as indicated by a marked increase in important intermediates of TCA cycle (citrate in serum and 2‐OG in kidney) in treatment groups as compared with LPS group. Thanks to the restoration of the TCA cycle by the treatments, other activated means of energy supply in LPS mice were also recovered to normal: HLJDD and its four component herbs significantly lowered the levels of the ketone bodies (acetoacetate, 3‐HB), creatine and creatinine (products of PCr after forming ATP for energy demand), breakdown products of ATP (adenosine and inosine).

Lipopolysaccharide‐induced kidney dysfunction via NF‐κB and MAPK activation, by excessive production of IL‐6, TNF‐α, iNOS, and COX‐2, producing perturbance in energy metabolism and oxidative stress. HLJDD and its four component herbs could effectively inhibit LPS‐induced AKI in mice and also markedly ameliorated disturbances in oxidative stress and energy metabolism induced by LPS. This study demonstrated that HLJDD exhibited the best anti‐AKI effect as a whole: with a stronger ability to improve survival rate, decrease Cr and BUN, accelerate Akt phosphorylation and enhance HO‐1 productions in LPS mice than the four component herbs. Specific effects of individual herb were found: RC, CP, and RS outperformed other treatments on inhibition of phosphorylation of Erk, p38, and p65, respectively, FG has exceptional ability to enhance the GSH level among all groups. In general, the four component herbs could complement to produce strong biological effects, and properties not even found in individual herbs, such as greatly activated Akt pathway. This study built a substantial basis for a systematic study on the underlying mechanisms of LPS‐induced AKI and based on which, to development new therapy.

## Author contributions

PL and L‐YK designed the study; PL, S‐TL, D‐QX, QZ, and LY conducted the experiments; M‐HY recorded the NMR data; PL and J‐SW analyzed the data; and PL wrote the paper.

## Supporting information


**Fig. S1.** HPLC chromatogram (254nm) of Standards (A), HLJDD (B) and its four herbs: RC (C), RS (D), CP (E), FG (F).

**Fig. S2.** HPLC‐Q TOF‐MS total ion chromatogram of HLJDD (A) and its four herbs: RC (B), RS (C), CP (D), FG (E).

**Table S1.** Compounds detected in the HLJDD obtained by HPLC‐Q‐TOF‐MS/MS.
**Table S2.** Compounds detected in the RC obtained by HPLC‐Q‐TOF‐MS/MS.
**Table S3.** Compounds detected in the RS obtained by HPLC‐Q‐TOF‐MS/MS.
**Table S4.** Compounds detected in the CP obtained by HPLC‐Q‐TOF‐MS/MS.
**Table S5.** Compounds detected in the FG obtained by HPLC‐Q‐TOF‐MS/MS.
