# Supplementary figures and images for: Protection by Huang‐Lian‐Jie‐Du decoction and its constituent herbs of lipopolysaccharide‐induced acute kidney injury
Source: FEBS Open Bio. 2017 Jan 11;7(2):221–36. doi: 10.1002/2211-5463.12178 (PMC5292670; doi:10.1002/2211-5463.12178)

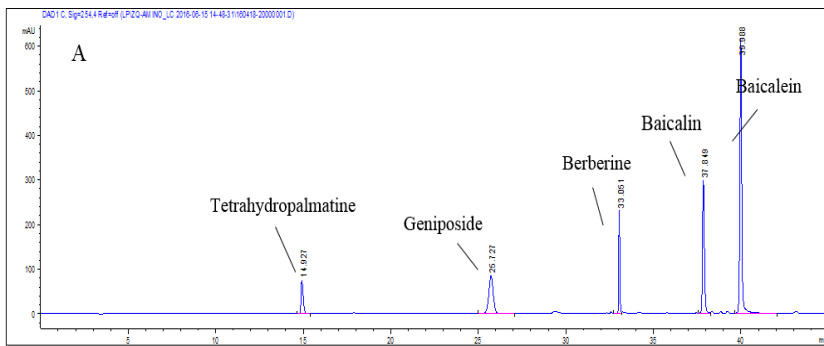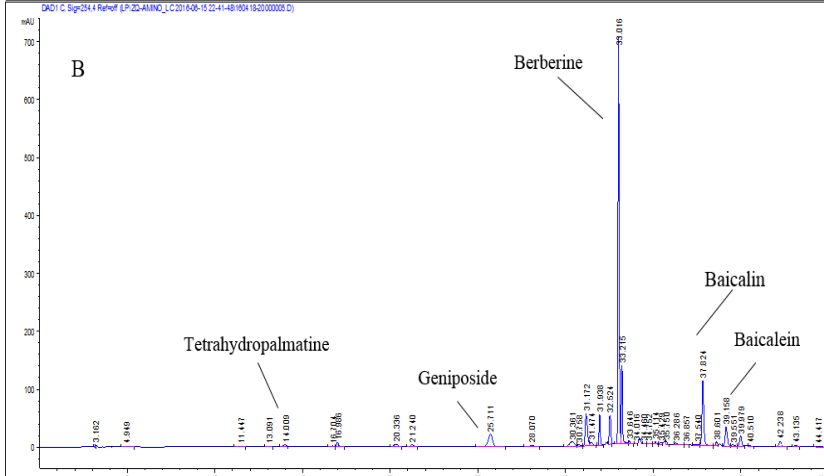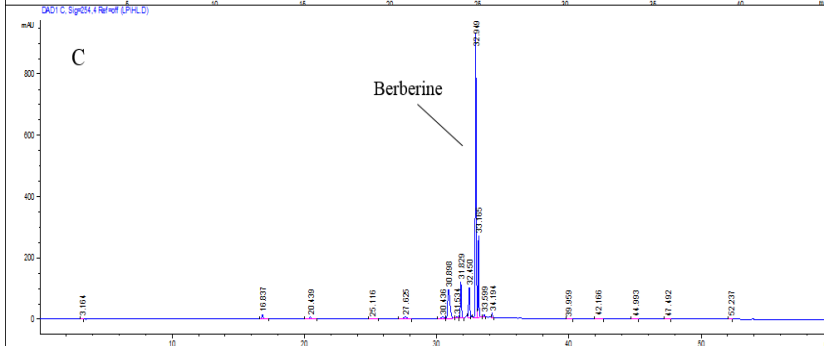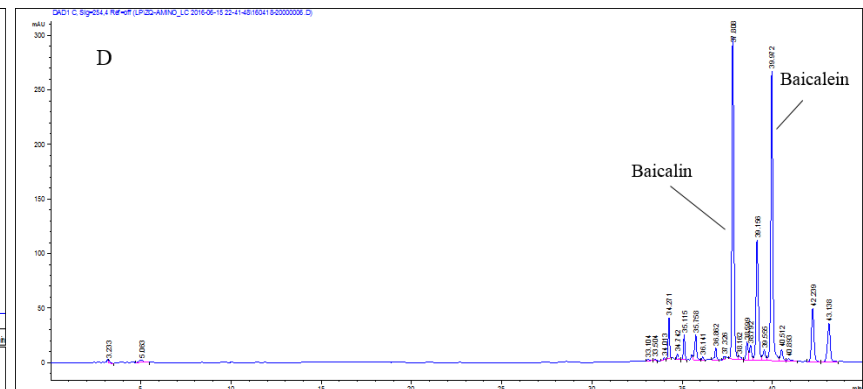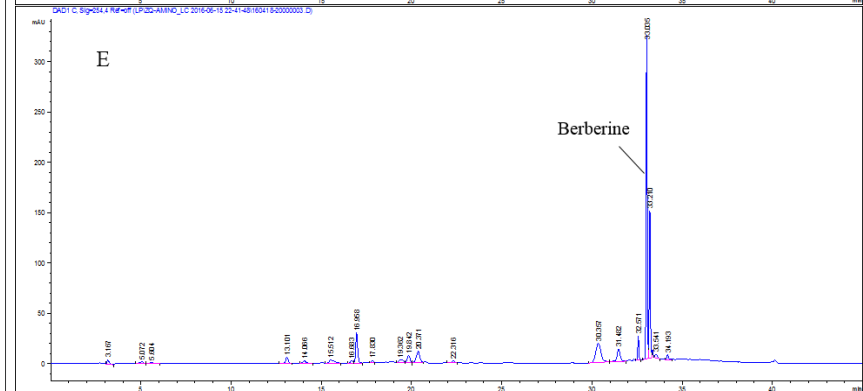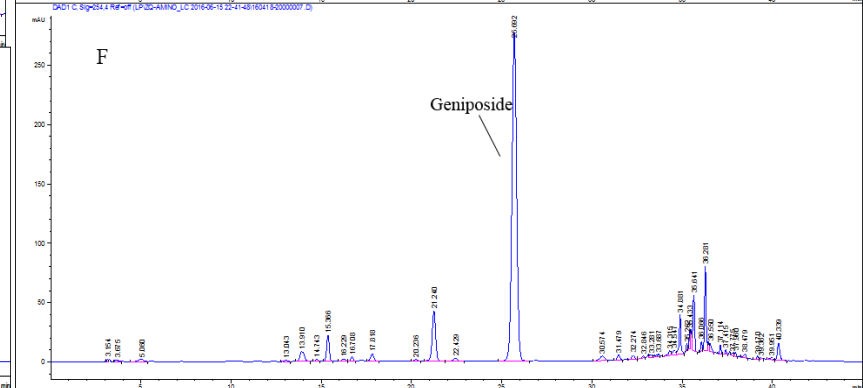

Supplement: Supplementary file 1 — Fig. S1. HPLC chromatogram (254nm) of Standards (A), HLJDD (B) and its four herbs: RC (C), RS (D), CP (E), FG (F). [file FEB4-7-221-s001.pdf]

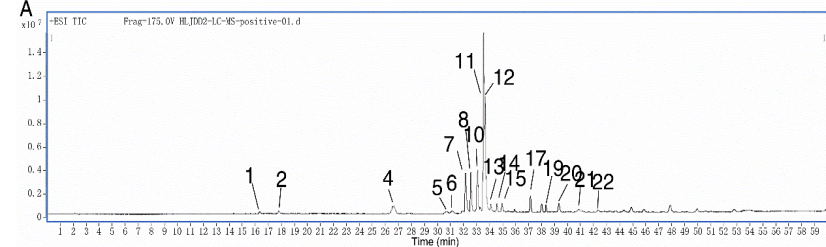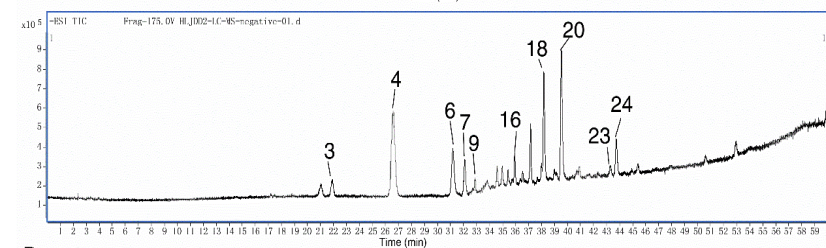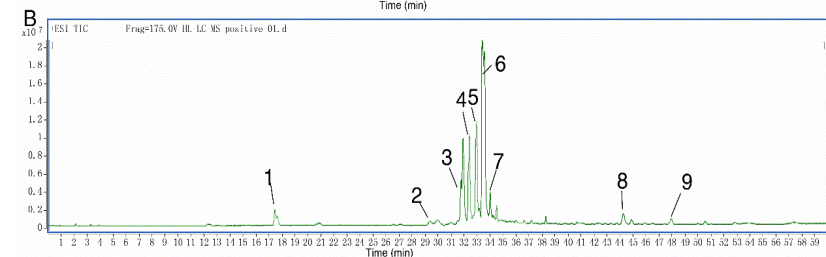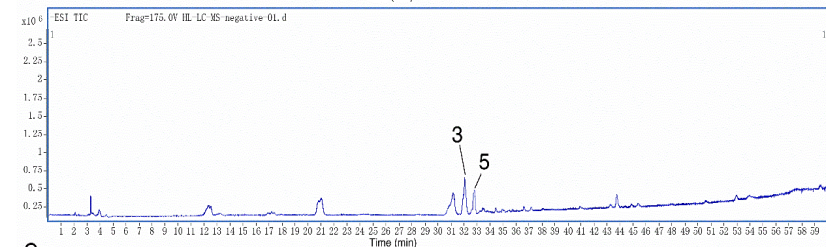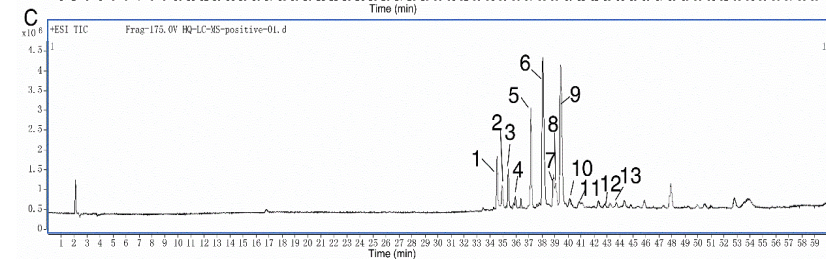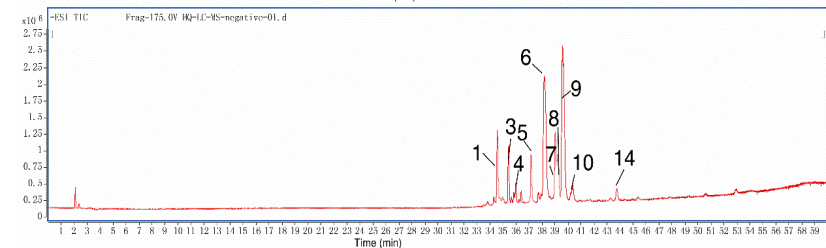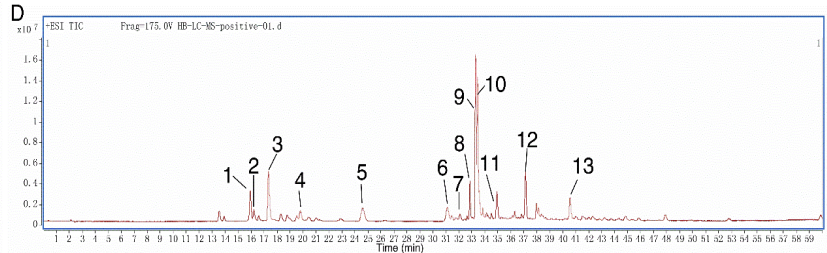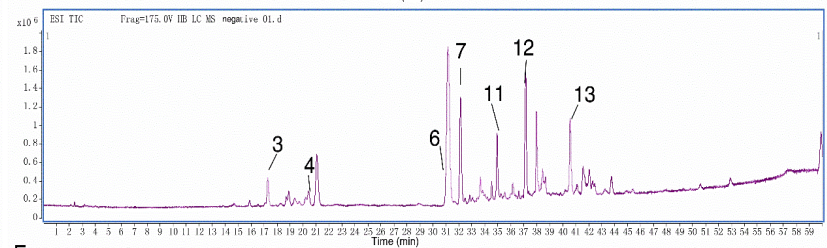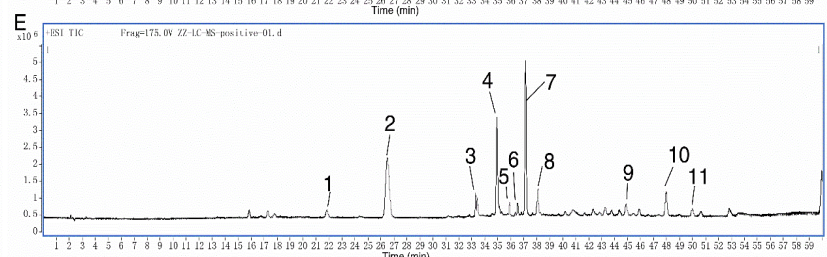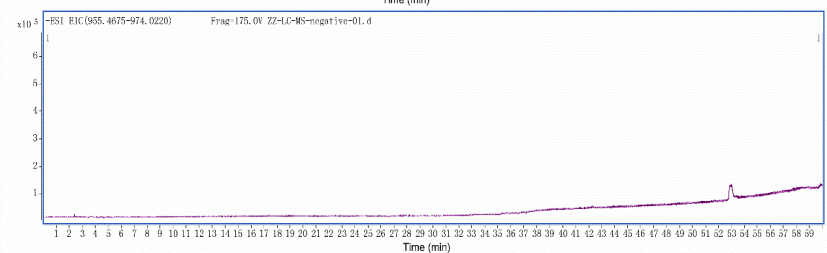

Supplement: Supplementary file 2 — Fig. S2. HPLC‐Q TOF‐MS total ion chromatogram of HLJDD (A) and its four herbs: RC (B), RS (C), CP (D), FG (E). [file FEB4-7-221-s002.pdf]
