# Supplementary material for: Protection by Huang‐Lian‐Jie‐Du decoction and its constituent herbs of lipopolysaccharide‐induced acute kidney injury
Source: FEBS Open Bio. 2017 Jan 11;7(2):221–36. doi: 10.1002/2211-5463.12178 (PMC5292670; doi:10.1002/2211-5463.12178)
Supplement: Supplementary file 3 — Table S1. Compounds detected in the HLJDD obtained by HPLC‐Q‐TOF‐MS/MS. Table S2. Compounds detected in the RC obtained by HPLC‐Q‐TOF‐MS/MS. Table S3. Compounds detected in the RS obtained by HPLC‐Q‐TOF‐MS/MS. Table S4. Compounds detected in the CP obtained by HPLC‐Q‐TOF‐MS/MS. Table S5. Compounds detected in the FG obtained by HPLC‐Q‐TOF‐MS/MS. [file FEB4-7-221-s003.docx]

**

Captions**:

Fig. S1 HPLC chromatogram (254nm) of Standards (A), HLJDD (B) and its four herbs: RC (C), RS (D), CP (E), FG (F).





Fig. S2 HPLC- Q TOF MS total ion chromatogram of HLJDD (A) and its four herbs: RC (B), RS (C), CP (D), FG (E).

**Tables**

Table S1 Compounds detected in the HLJDD obtained by HPLC-Q-TOF-MS/MS.

| Peak No | t_R_(min) | M | Molecular Structure | Mode | Compound identity |
| --- | --- | --- | --- | --- | --- |
| 1 | 16.325 | 341.1629 | C_20_H_23_NO_4_ | positive | Phellodendrine |
| 2 | 17.765 | 341.1427 | C_20_H_23_NO_4_ | positive | Magnoflorine |
| 3 | 21.827 | 549.1716 | C_23_H_34_O_15_ | negative | genipin-1-β-D-gentiobioside |
| 4 | 26.553 | 388.1189 | C_17_H_24_O_10_ | both | Geniposide |
| 5 | 30.665 | 321.1009 | C_19_H_15_NO_4_ | positive | Berberubine |
| 6 | 31.128 | 368.1103 | C_17_H_20_O_9_ | both | 3-ofernloyquini acid |
| 7 | 32.154 | 319.0846 | C_19_H_13_NO_4_ | both | Coptisine |
| 8 | 32.569 | 335.1156 | C_20_H_17_NO_4_ | positive | Epiberberine |
| 9 | 32.978 | 337.1315 | C_20_H_19_NO_4_ | negative | Jatrorrhizine |
| 10 | 33.027 | 337.1316 | C_20_H_19_NO_4_ | positive | Columbamine |
| 11 | 33.491 | 352.1554 | C_21_H_21_NO_4_ | positive | Palmatine |
| 12 | 33.756 | 335.1131 | C_20_H_17_NO_4_ | positive | Berberine |
| 13 | 34.059 | 349.1312 | C_21_H_19_NO_4_ | positive | Unknown |
| 14 | 34.522 | 527.1584 | C_30_H_25_NO_8_ | positive | Unknown |
| 15 | 34.877 | 452.3363 | C_23_H_48_O_8_ | positive | Sorbitan |
| 16 | 35.894 | 696.2254 | C_32_H_40_O_17_ | negative | Phelloside |
| 17 | 37.078 | 678.5047 | C_40_H_70_O_8_  /C_36_H_66_N_6_O_6_ | positive | Unknown |
| 18 | 37.949 | 446.0848 | C_21_H_18_O_11_ | negative | Baicalin |
| 19 | 38.536 | 446.0924 | C_21_H_18_0_11_ | positive | Baicalinisomer |
| 20 | 39.463 | 460.1003 | C_22_H_20_O_11_ | both | Oroxyloside |
| 21 | 40.810 | 374.1001 | C_19_H_18_O_8_ | positive | 5,7-Dihydroxy-6,8-dimethoxyflavone |
| 22 | 42.630 | 284.0568 | C_16_H_12_O_5_ | positive | Oroxylin A |
| 23 | 43.610 | 284.0766 | C_16_H_12_O_5_ | negative | Wogonin |
| 24 | 43.752 | 271.2098 | C_15_H_10_O_5_ | negative | Baicalein |
| 25 | 47.929 | 276.1727 | C_17_H_24_O_3_ | negative | Unknown |

Table S2 Compounds detected in the RC obtained by HPLC-Q-TOF-MS/MS.

| Peak No | t_R_(min) | M | Molecular Structure | Mode | Compound identity |
| --- | --- | --- | --- | --- | --- |
| 1 | 17.452 | 241.1699 | C_20_H_23_NO_4_ | positive | Phellodendrine |
| 2 | 29.362 | 321.1004 | C_19_H_15_NO_4_ | positive | Berberubine |
| 3 | 31.810 | 319.0857 | C_19_H_13_NO_4_ | both | Coptisine |
| 4 | 32.392 | 335.117 | C_20_H_17_NO_4_ | positive | Epiberberine |
| 5 | 32.851 | 337.1329 | C_20_H_19_NO_4_ | both | Jatrorrhizine |
| 6 | 33.407 | 336.1232 | C_20_H_17_NO_4_ | positive | Berberine |
| 7 | 33.609 | 349.1314 | C_21_H_19_NO_4_ | positive | Unknown |
| 8 | 44.2110 | 352.1188 | C_20_H_17_NO_5_ | positive | Palmatine |
| 9 | 47.929 | 276.1727 | C17H24O3 | positive | Unknown |

Table S3 Compounds detected in the RS obtained by HPLC-Q-TOF-MS/MS.

| Peak No | t_R_(min) | M | Molecular Structure | Mode | Compound identity | | |
| --- | --- | --- | --- | --- | --- | --- | --- |
| 1 | 34.517 | 549.1603 | C_26_H_28_O_13_ | both | Chrysin-6-C-arabinoside-8-C-glucoside | | |
| 2 | 34.904 | 453.3441 | C_23_H_48_O_8_ | positive | Unknown | | |
| 3 | 35.341 | 549.1598 | C_26_H_18_O_13_ | both | Chrysin-6-C-glucoside-8-C-arabinoside | | |
| 4 | 35.925 | 549.1609 | C_26_H_18_O_13_ | both | Chrysin-6-C-glucoside-8-C-arabinoside | | |
| 5 | 37.078 | 678.5047 | C_40_H_70_O_8_  /C_36_H_66_N_6_O_6_ | both | Unknown |  |  |
| 6 | 38.042 | 446.0944 | C_21_H_18_O_11_ | both | Baicalin | | |
| 7 | 38.871 | 446.0922 | C_21_H_18_O_11_ | both | Baicalinisomer | | |
| 8 | 39.018 | 431.0978 | C_21_H_18_O_10_ | both | Chrysin 7-glucuronide | | |
| 9 | 39.384 | 461.1090 | C_22_H_20_O_11_ | both | Oroxyloside | | |
| 10 | 40.066 | 461.1080 | C_22_H_20_O_11_ | both | Oroxylosideisomer | | |
| 11 | 40.810 | 374.1001 | C_19_H_18_O_8_ | positive | 5,7-Dihydroxy-6,8-dimethoxyflavone | |  |
| 12 | 42.630 | 284.0568 | C_16_H_12_O_5_ | positive | Oroxylin A | |  |
| 13 | 43.610 | 284.0766 | C_16_H_12_O_5_ | positive | Wogonin | |  |
| 14 | 43.752 | 271.2098 | C_15_H_10_O_5_ | negative | Baicalein | |  |

Table S4 Compounds detected in the CP obtained by HPLC-Q-TOF-MS/MS.

| Peak No | t_R_(min) | M | Molecular Structure | Mode | Compound identity |
| --- | --- | --- | --- | --- | --- |
| 1 | 15.862 | 342.1628 | C_20_H_23_NO_4_ | positive | Phellodendrine |
| 2 | 16.173 | 273.1632 | C_16_H_19_NO_3_ | positive | Unknown |
| 3 | 17.303 | 342.1632 | C_20_H_23_NO_4_ | both | Phellodendrine |
| 4 | 19.818 | 356.1854 | C_21_H_25_NO_4_ | both | Tetrahydropalmatine |
| 5 | 24.500 | 356.1854 | C_21_H_25_NO_4_ | positive | Tetrahydropalmatine |
| 6 | 31.026 | 368.1103 | C_17_H_20_O_9_ | both | 3-ofernloylguini acid |
| 7 | 32.052 | 338.1389 | C_20_H_20_NO_4_ | both | Jatrorrhizine |
| 8 | 32.834 | 337.1315 | C_20_H_19_NO_4_ | positive | Unknown |
| 9 | 33.285 | 336.1252 | C_20_H_18_NO_4_ | positive | Berberine |
| 10 | 33.798 | 352.1545 | C_21_H_22_NO_4_ | positive | Palmatine |
| 11 | 34.829 | 453.3434 | C_23_H_48_O_8_ | positive | Unknown |
| 12 | 37.088 | 629.5117 | C_36_H_66_N_6_O_6_ | both | Unknown |
| 13 | 40.531 | 454.1994 | C_26_H_30_O_7_ | both | Obacunone |

Table S5 Compounds detected in the FG obtained by HPLC-Q-TOF-MS/MS.

| Peak No | t_R_(min) | M | Molecular Structure | Mode | Compound identity | |
| --- | --- | --- | --- | --- | --- | --- |
| 1 | 21.767 | 549.1716 | C_23_H_34_O_15_ | positive | Genipin-1-β-D-gentiobioside | |
| 2 | 26.443 | 388.1189 | C_17_H_24_O_10_ | positive | Geniposide | |
| 3 | 33.362 | 695.2070 | C_18_H_24_O_7_ | positive | 6’’-O-trans-p-eoumaroylge-nipin gentiobioside | |
| 4 | 34.934 | 351.1543 | C_23_H_48_O_8_ | positive | Penicimenolide B | |
| 5 | 35.796 | 452.3356 | C_32_H_40_O_12_ | positive | β-​D-​Xylofuranoside | |
| 6 | 36.494 | 975.3599 | C_44_H_64_O_24_ | positive | Crocin-1 | |
| 7 | 37.105 | 655.5049 | C_38_H_72_O_8_ | positive | β-​D-​Glucopyranose | |
| 8 | 44.935 | 391.2042 | C_22_H_32_O_6_ | positive | 2-​Butenoic acid | |
| 9 | 45.383 | 223.1411 | C_13_H_20_O_3_ | positive | Unknown | |
| 10 | 47.929 | 276.1727 | C_17_H_24_O_3_ | positive | Unknown |  |
| 11 | 49.895 | 203.0788 | C_10_H_14_O_3_ | positive | Furancarboxylic acid | |
